# Outcomes of sentinel node biopsy according to MRI response in an association with the subtypes in cN1–3 breast cancer after neoadjuvant systemic therapy, multicenter cohort study

**DOI:** 10.1186/s13058-024-01807-8

**Published:** 2024-04-17

**Authors:** Soong June Bae, Jung Whan Chun, Sae Byul Lee, Jai Min Ryu, Seok Jin Nam, Joon Jeong, Hyung Seok Park, Sung Gwe Ahn

**Affiliations:** 1 Department of Surgery, Gangnam Severance Hospital, Yonsei University College of Medicine, Seoul, Republic of Korea; 2 Institute for Breast Cancer Precision Medicine, Gangnam Severance Hospital, Yonsei University College of Medicine, Seoul, Republic of Korea; 3 Department of Surgery, Asan Medical Center, Seoul, Republic of Korea; 4 Present Address: Department of Surgery, Seoul National University Hospital, Seoul, Republic of Korea; 5 Department of Surgery, Samsung Medical Center, Seoul, Republic of Korea; 6 Department of Surgery, Yonsei Cancer Center, Yonsei University College of Medicine, Seoul, Republic of Korea

**Keywords:** Breast neoplasm, Breast MRI, Neoadjuvant systemic therapy, Axilla, Sentinel lymph node biopsy

## Abstract

**Background:**

This study investigated the feasibility of sentinel lymph node biopsy (SLNB) after neoadjuvant systemic therapy (NAST) in patients with initially high nodal burden.

**Methods:**

In the multicenter retrospective cohort, 388 individuals with cN1–3 breast cancer who underwent NAST and had SLNB followed by completion axillary lymph node dissection were included. In an external validation cohort, 267 patients with HER2+ or triple-negative breast cancer (TNBC) meeting similar inclusion criteria were included. Primary outcome was the false-negative rates (FNRs) of SLNB according to the MRI response and subtypes. We defined complete MRI responders as patients who experienced disappearance of suspicious features in the breast and axilla after NAST.

**Results:**

In the multicenter retrospective cohort, 130 (33.5%) of 388 patients were of cN2-3, and 55 (14.2%) of 388 patients showed complete MRI responses. In hormone receptor-positive HER2− (n = 207), complete and non-complete responders had a high FNRs (31.3% [95% CI 8.6–54.0] and 20.9% [95% CI 14.1–27.6], respectively). However, in HER2+ or TNBC (n = 181), the FNR of complete MRI responders was 0% (95% CI 0–0), whereas that of non-complete responders was 33.3% (95% CI 20.8–45.9). When we validated our findings in the external cohort with HER2+ or TNBC (n = 267), of which 34.2% were cN2-3, the FNRs of complete were 7.1% (95% CI 0–16.7).

**Conclusions:**

Our findings suggest that SLNB can be a reliable option for nodal status evaluation in selected patients who have responded well to NAST, especially in HER2+ and TNBC patients who show a complete MRI response.

**Supplementary Information:**

The online version contains supplementary material available at 10.1186/s13058-024-01807-8.

## Background

Neoadjuvant systemic therapy (NAST) is widely adopted as the standard of care for patients with clinical stage II–III breast cancer [1–3]. Advancements in NAST, such as human epidermal growth factor receptor 2 (HER2)-targeted therapy or immune-check point blockade, have increased the pathologic complete response (pCR) rate, even in patients with locally advanced breast cancer [4–6]. Additionally, NAST has been shown to increase the breast-conserving surgery rate and decrease the axillary lymph node dissection (ALND) rate by down-staging breast cancer [7]. In this context, many investigators have evaluated the feasibility of sentinel lymph node biopsy (SLNB) after NAST for clinically node-positive (cN1–3) breast cancer at initial diagnosis [8–12]. Although previous pivotal trials, such as ACOSOG Z1071 and SENTINA, showed that the false-negative rates (FNR) of SLNB were higher than 10% and were not acceptable in the general population, the subgroup analyses of these trials suggested that SLNB could be considered in selected patients with negative SLNB results on examination of three or more SLNs [10, 11]. Additionally, the SN FNAC study showed that SLNB may be performed if tumor cells are not found in resected SLNs on immunohistochemistry (IHC) examination [12]. These results permit less extensive axillary surgery in patients with nodal involvement at initial diagnosis who received NAST [13, 14].

Meanwhile, in previous landmark trials, most patients had a relatively low nodal burden of cN1, which was determined by physical examination rather than with radiologic modalities [10–12]. In addition, post-NAST imaging studies, including breast magnetic resonance imaging (MRI), are increasingly important for assessing post-NAST axillary staging [15–17]. Several studies suggest that breast MRI or an integrated model using radiologic and clinical information is helpful in evaluating post-NAST axillary nodal response [15–19]. Nevertheless, there is limited evidence that SLNB could be performed in patients with node-positive breast cancer, including those with high axillary metastatic volume who achieved complete radiologic response after NAST. Furthermore, whether breast MRI enables the accurate assessment of axillary response after NAST in cN1–3 breast cancer remains unclear [20–22].

We sought to determine whether SLNB could be a reliable option for evaluating nodal status in patients who had responded well to NAST, even if they had initially presented with a high nodal burden. The study investigated the outcomes of SLNB followed by axillary lymph node dissection (ALND) in this patient population, taking into account the response to NAST and the breast cancer subtype. Additionally, we assessed whether additional ALND can be omitted in highly selected patients with 1–2 metastatic SLNs after NAST, which indicates a low residual volume of axillary metastasis.

## Methods

### Study population

The study protocol was reviewed and approved by the institutional review board of each institution (IRB no. 3-2022-0362) and adhered to the tenets of the Declaration of Helsinki. The requirement for written informed consent was waived owing to the retrospective study design. In addition, this work has been reported in line with STROCSS criteria (NCT05779982) [23].

We used databases from four institutions (Gangnam Severance Hospital [GSH], Yonsei Cancer Center [YCC], Asan Medical Center [AMC], and Samsung Medical Center [SMC]) to retrospectively identify patients diagnosed with breast cancer who received NAST followed by curative surgery between January 2013 and December 2018. Inclusion criteria were as follows: (i) cN1–3 breast cancer at initial presentation, (ii) SLNB followed by additional ALND, and (iii) breast MRI performed at baseline and post-NAST. We enrolled patients with cN1–3 breast cancer at initial presentation whose axillary nodal involvement was revealed by breast MRI. The clinical nodal stage was determined based on findings from breast MRI according to the American Join Committee on Cancer guidelines (7th edition). In addition, we included patients who had low suspicious lymph nodes on MRI, which was pathologically confirmed by ultrasonography-guided biopsy.

**Fig. 1:**
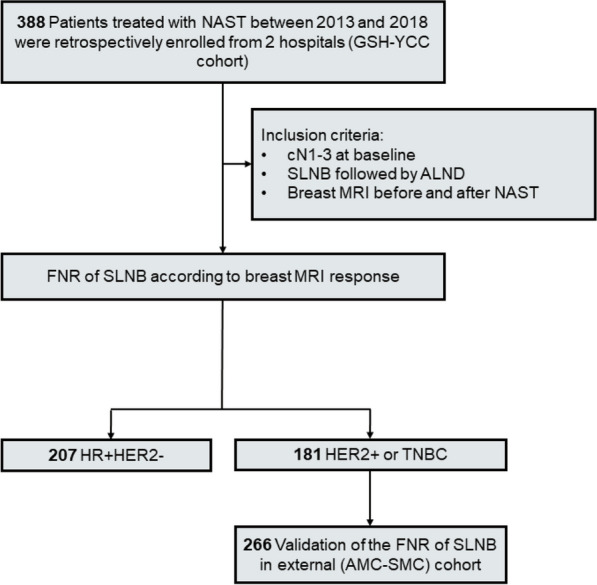
Study diagram

For axillary surgery, the patients underwent SLNB followed by ALND. SLNB was performed using a radioactive marker, blue dye, or both (dual tracers). We defined axillary lymph nodes with more than 10% of the hottest node’s radioactivity or stained with blue dye as sentinel lymph nodes [24]. The patients were classified into three subtypes using three IHC markers: estrogen receptor (ER), progesterone receptor (PR), and human epidermal growth factor receptor-2 (HER2); hormone receptor HR+ HER2− and HER2+; and triple-negative breast cancer (TNBC) subtypes. We then performed our analyses in two groups stratified by subtype: (i) HR+ HER2− as NAST-non-sensitive and (ii) HER2+ or TNBC as NAST-sensitive. Patients with all breast cancer subtypes were included in the GSH-YCC cohort, whereas those with HR+ HER2− breast cancer were excluded from the AMC-SMC cohort. We first analyzed the FNR of SLNB according to NAST response in the subgroups stratified by subtypes in GSH-YCC (discovery cohort), and we then verified the findings in HER2+ or TNBC in AMC-SMC (validation cohort). All patients with HR+ HER2− breast cancer or TNBC received anthracycline- or taxane-based chemotherapy for NAST. All patients with HER2+ breast cancer received anthracycline- or taxane-based chemotherapy plus HER2−targeted therapies, such as trastuzumab with/without pertuzumab, during the NAST. Finally, 388 patients from the GSH-YCC cohort and 266 patients from the AMC-SMC cohort were included in the present study (Fig. 1). The radiologic response to NAST was assessed using post-treatment MRI.

We reviewed clinicopathologic data, such as age at diagnosis, histologic grade, ER status, PR status, HER2 status, Ki-67 levels, clinical T stage, and clinical N stage. The clinical T and N stages were evaluated on the basis of pre-treatment multimodal imaging studies, including breast MRI and ultrasonography with/without positron emission tomography-computed tomography, which were determined according to the anatomical stage based on the American Joint Committee on Cancer guidelines (eighth edition). All pathological data were obtained from core needle biopsy samples before NAST. Pathologic complete response (pCR) was defined as the absence of invasive tumors in surgical samples, including the breast and axilla tumors. Cases with ypN0i+ were regarded as non-pCR.

### Radiologic response

Breast MRI was performed at the time of diagnosis and after completion of NAST in all patients. All bilateral axial images were acquired from patients in the prone position. The fat-suppressed T1-weighted and subtraction axial images of the ALN and index breast obtained from early post-contrast were interpreted by expert radiologists at each institution. We determined the ALN and breast lesion status based on the interpretation of the breast MRI from the radiologic reports. In general, the ALN was assessed as positive if suspicious features, such as irregular margins, round shapes, eccentric cortical thickening, and loss of fatty hilum, were observed. Breast lesions were classified as malignant if a higher enhancement was present with mass- or non-mass-like lesions than with breast parenchymal enhancement or contralateral breast (Additional file 1: Figs. S1, S2). We defined a complete MRI responder as the absence of suspicious features in both the breast and ALNs on breast MRI after NAST, with reference to breast MRI at baseline (Additional file 1: Fig. S1) [25].

### Statistical analyses

To determine the feasibility of SLNB in this study, we evaluated the FNR of SLNB. The FNR was calculated as the number of patients with negative SLNs who had residual disease in the rest of ALNs divided by the total number of patients with residual disease in either the SLNs, the rest of the ALNs, or both: FN/true-positive + FN. We compared the FNR of the SLNB according to the radiologic response measured using breast MRI in each subtype. We further investigated the FNR in subgroups stratified by clinical nodal stage. Additionally, we investigated cases with 1–2 metastatic SLNs who had no additional metastatic ALNs through the completion of ALND. Continuous variables were compared using the Student’s t-test, and discrete variables were compared using the Chi-square test or Fisher’s exact test. All tests were two-sided, and *P* values less than 0.05 were considered statistically significant. All analyses were performed using SPSS version 25 software (SPSS, Armonk, NY, USA).

## Results

### Baseline characteristics

**Fig. 2:**
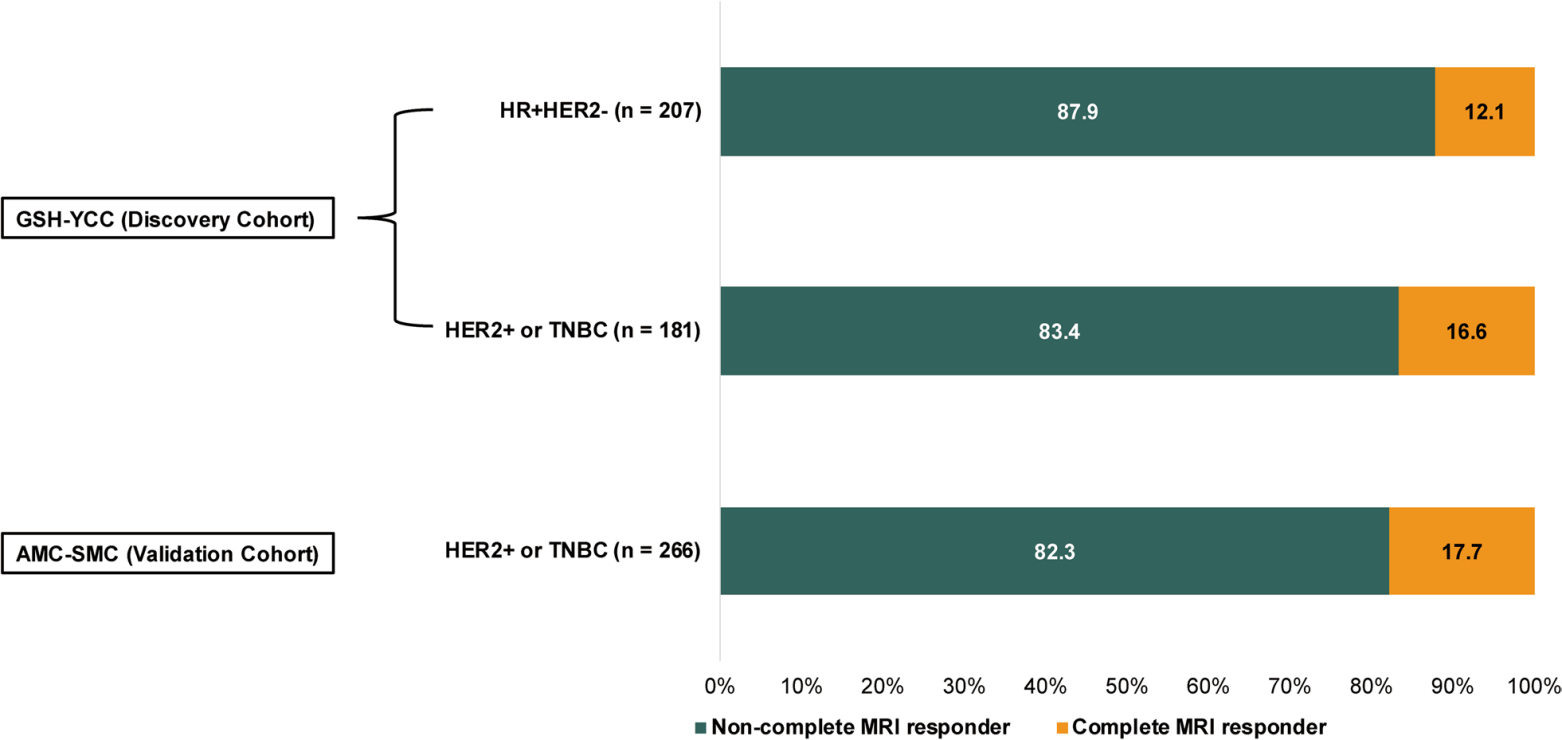
The rates of complete MRI response. In the GSH-YCC cohort, 207 patients had HR+ HER2− breast cancer and 181 patients had HER2+ breast cancer (n = 181). Of the 388 patients, 55 (14.2%) were complete MRI responders, 25 (12.1%) of 207 patients in HR+ HER2−, and 30 (16.6%) of 181 patients in HER2+ or TNBC. In the validation cohort (AMC-SMC) consisting of 288 patients with HER2+ or TNBC, 47 (17.7%) patients achieved complete MRI response after NAST. HR, hormone receptor; HER2, human epidermal growth factor receptor 2; TNBC, triple-negative breast cancer

The discovery cohort consisted of 388 women who met the inclusion criteria and were identified from the GSH-YCC cohort. Of these, 207 patients had HR+ HER2− breast cancer and 181 patients had HER2+ breast cancer (n = 75) or TNBC (n = 106). Of the 388 patients, 55 (14.2%) were complete MRI responders, 25 (12.1%) of 207 patients in HR+ HER2−, and 30 (16.6%) of 181 patients in HER2+ or TNBC (Fig. 2). Among the patients with HR+ HER2− breast cancer, the complete MRI responders had a higher proportion of PR- tumors than did the non-complete MRI responders (40.0% vs. 22.5%, *P* = 0.057, Additional file 1: Table S1). Additionally, 78 (37.7%) of the 207 patients had cN2-3 at baseline; the proportion of cN2-3 did not differ according to the MRI response. The number of removed ALNs was 15 (range, 10–33); the complete MRI responders had lower number of removed ALNs than the non-complete MRI responders (13 [range, 10–30] vs. 16 [range, 10–33], *P* = 0.049, Additional file 1: Table S1). In patients with HER2+ or TNBC, complete MRI responders were more likely to have clinical tumor stage I than were non-complete MRI responders (10.0% vs. 2.7%, *P* = 0.040, Table 1). Moreover, 52 (28.7%) of the 181 patients had cN2-3 at baseline. The number of removed ALNs was 15 (range, 10–38); the number of removed ALNs was lower in complete MRI responders than in non-complete MRI responders (13 [range, 10–28] vs. 16 [range, 10–38], *P* = 0.015). As in the HR+ HER2− breast cancer, the proportion of cN2-3 did not differ according to the MRI response (Table 1). Among the 75 patients with HER2+ breast cancer, 33 (44.0%) received neoadjuvant chemotherapy plus trastuzumab, while 42 (56.0%) received neoadjuvant chemotherapy plus trastuzumab and pertuzumab (Table 1).

**Table 1:** Baseline characteristics of patients with HER2+ and TNBC breast cancer according to MRI response

	GSH-YCC (discovery cohort)				AMC-SMC (validation cohort)			
Variables	Non-complete MRI responders (N = 151)	Complete MRI responders (N = 30)	Total (N = 181)	*P* value	Non-complete MRI responders (N = 219)	Complete MRI responders (N = 47)	Total (N = 266)	*P* value
*Age at diagnosis, y*				0.760				0.102
< 50	100 (66.2)	19 (63.3)	119 (65.7)		88 (40.2)	25 (53.2)	113 (42.5)	
≥ 50	51 (33.8)	11 (36.7)	62 (34.3)		131 (59.8)	22 (46.8)	153 (57.5)	
*Subtype*				0.324				0.398
HER2+	65 (43.0)	10 (33.3)	75 (41.4)		122 (55.7)	23 (48.9)	145 (54.5)	
TNBC	86 (57.0)	20 (66.7)	106 (58.6)		97 (44.3)	24 (51.1)	121 (45.5)	
*Pathologically confirmed lymph node*^*a*^				0.501				0.682
Yes	96 (63.6)	21 (70.0)	117 (64.6)		196 (89.5)	43 (91.5)	239 (89.8)	
No	55 (36.4)	9 (30.0)	64 (35.4)		23 (10.5)	4 (8.5)	27 (10.2)	
Removed lymph node^b^	16 (10–38)	13 (10–28)	15 (10–38)	0.015	10 (5–34)	10 (5–31)	10 (5–34)	0.993
*Histologic grade*^*c*^				0.108				
1 or 2	68 (62.4)	14 (82.4)	82 (65.1)					
3	41 (37.6)	3 (17.6)	44 (34.9)					
*Ki-67*^*a*^				0.861				
< 14%	34 (27.9)	6 (26.1)	40 (27.6)					
≥ 14%	88 (72.1)	17 (73.9)	105 (72.4)					
*Clinical T stage*				0.040				0.015
1	4 (2.7)	3 (10.0)	7 (3.9)		4 (1.8)	4 (8.5)	8 (3.0)	
2	95 (62.9)	22 (73.3)	117 (64.6)		147 (67.1)	24 (51.1)	171 (64.3)	
≥ 3	52 (34.4)	5 (16.7)	57 (31.5)		68 (31.1)	19 (40.4)	87 (32.7)	
*Clinical N stage*				0.945				0.320
1	100 (66.2)	19 (63.3)	119 (65.7)		147 (67.1)	28 (59.6)	175 (65.8)	
2	15 (9.9)	3 (10.0)	18 (9.9)		20 (9.1)	3 (6.4)	23 (8.6)	
3	36 (23.8)	8 (26.7)	44 (24.3)		52 (23.7)	16 (34.0)	68 (25.6)	
*Breast operation*				0.042				0.095
Breast-conserving surgery	70 (46.4)	20 (66.7)	90 (49.7)		99 (45.2)	15 (31.9)	114 (42.9)	
Mastectomy	81 (53.6)	10 (33.3)	91 (50.3)		120 (54.8)	32 (68.1)	152 (57.1)	
*Neoadjuvant systemic therapy regimen*				0.373				0.081
Chemotherapy	86 (57.0)	20 (66.7)	106 (58.6)		97 (44.3)	24 (51.1)	121 (45.5)	
Chemotherapy + Trastuzumab	27 (17.9)	6 (20.0)	33 (18.2)		103 (47.0)	15 (31.9)	118 (44.4)	
Chemotherapy + Trastuzumab + Pertuzumab	38 (25.2)	4 (13.3)	42 (23.2)		19 (8.7)	8 (17.0)	27 (10.2)	

Data are reported as the number (percentage) of patients unless otherwise indicated

*MRI* magnetic resonance imaging, *HER2* human epidermal growth factor receptor 2, *TNBC* triple-negative breast cancer

^a^Patients with non-pathologically confirmed lymph nodes and radiologically suspicious lymph nodes are included, and nodal staging work-up is assessed using multiple imaging studies

^b^Data are reported as the number (range) of removed axillary lymph nodes

^c^Missing values. However, these values have not been assessed in the validation cohort

The validation cohort consisted of 288 women with HER2+ or TNBC identified from the AMC-SMC database. Within this cohort, 47 (17.7%) patients achieved complete MRI response after NAST (Fig. 2), and 91 (34.2%) had cN2-3 stage disease before NAST (Table 1). The number of removed ALNs was 10 (range, 10–34); it was not different according to the MRI response. Among the 145 patients with HER2+ breast cancer, 118 (81.4%) received neoadjuvant chemotherapy plus trastuzumab, while 27 (18.6%) received neoadjuvant chemotherapy plus trastuzumab and pertuzumab (Table 1). Similar to the GSH-YCC cohort, complete MRI responders were more likely to have clinical tumor stage I than were non-complete MRI responders (8.5% vs. 1.8%, *P* = 0.015, Table 1). The other clinicopathologic factors did not differ according to the MRI response.

### False-negative rate of sentinel lymph node biopsy according to treatment response

First, we investigated the FNRs of SLNB according to MRI response in each subtype in the discovery cohort (Table 2). Among HR+ HER2− breast cancer patients from the GSH-YCC cohort, FNRs were much higher than 10%, regardless of the MRI response: FNRs were 31.3% (95% confidence interval [CI] 8.6%–54.0%) (5 of 16 patients) in the complete MRI responders and 20.9% (95% CI 14.1%–27.6%) (29 of 139 patients) in non-complete MRI responders. By contrast, FNRs differed according to MRI response in HER2+ or TNBC in the GSH-YCC cohort. The FNR of SLNB in complete MRI responders was 0% (0 of 6 patients), whereas it was 33.3% (95% CI 20.8%–45.9%) (18 of 54 patients) in non-complete MRI responders. Among non-complete MRI responders, the FNRs were 23.8% (95% CI 6–42.0) (5 or 21 patients) in patients with cN2-3 disease at baseline and 39.4% (95% CI 22.7–56.1) in patients with cN1 disease at baseline (Table 2).

**Table 2:** False-negative rate of sentinel lymph node biopsy according to radiologic response

Cohort	Subtypes	Treatment response	All patients				cN2–3				cN1			
GSH-YCC	HR+ HER2−			ALN+	ALN−	FNR^a^		ALN+	ALN−	FNR^a^		ALN+	ALN−	FNR^a^
		Complete MRI response	SLN+	11	0	31.3% (8.6–54.0)	SLN+	2	0	50.0% (1.0–99.0)	SLN+	9	0	25.0% (0.5–49.5)
			SLN−	5	9		SLN−	2	4		SLN−	3	5	
		Non-complete MRI response	SLN+	110	0	20.9% (14.1–27.6)	SLN+	44	0	17.0% (6.9–27.1)	SLN+	66	0	23.3% (14.3–32.2)
			SLN−	29	43		SLN−	9	7		SLN−	20	36	

	HER2+ or TNBC			ALN+	ALN−	FNR^a^		ALN+	ALN−	FNR^a^		ALN+	ALN−	FNR^a^
		Complete MRI response	SLN+	6	0	0.0%	SLN+	0	0	0.0%	SLN+	6	0	0.0%
			SLN−	0	24		SLN−	0	11		SLN−	0	13	
		Non-complete MRI response	SLN+	36	0	33.3% (20.8–45.9)	SLN+	16	0	23.8% (6.0–42.0)	SLN+	20	0	39.4% (22.7–56.1)
			SLN−	18	97		SLN−	5	30		SLN−	13	67	

AMC-SMC	HER2+ or TNBC			ALN+	ALN−	FNR^a^		ALN+	ALN−	FNR^a^		ALN+	ALN−	FNR^a^
		Complete MRI response	SLN+	26	0	7.1% (0–16.7)	SLN+	14	0	6.7% (0–19.3)	SLN+	12	0	7.7% (0–22.2)
			SLN−	2	19		SLN−	1	4		SLN−	1	15	
		Non-complete MRI response	SLN+	105	0	11.8% (6.0–17.6)	SLN+	47	0	9.6% (1.6–17.6)	SLN+	58	0	13.4% (5.3–21.6)
			SLN−	14	100		SLN−	5	20		SLN−	9	80	

*ALN* axillary lymph node, *cN* clinical nodal stage, *FNR* false negative rate, *HER2* human epidermal growth factor receptor 2, *HR* hormone receptor, *MRI* magnetic resonance imaging, *SLN* sentinel lymph node, *TNBC* triple-negative breast cancer

^a^Data in parentheses are 95% confidence intervals

According to the results from patients with HER2+ or TNBC in the discovery cohort, we validated our findings in the independent cohort (AMC-SMC cohort). We explored the FNRs of SLNB according to MRI response in the validation cohort, consisting of patients with HER2+ or TNBC who met the same inclusion criteria as those of the discovery cohort. Moreover, 2 of 28 patients had FN SLNB with an FNR of 7.1% (95% CI 0–16.7) in the MRI responders, whereas 14 of 119 patients had FN SLNB with an FNR of 11.8% (95% CI 6.0–17.6) in non-complete MRI responders (Table 2). Similar results were observed when we analyzed the FNRs according to the clinical nodal stage at baseline (Table 2).

### Non-SLN metastasis in patients with HER2+ or TNBC who had 1–2 metastatic SLNs

To address whether ALND could be omitted in patients with 1–2 metastatic SLNs, we investigated additional non-SLN metastases in patients with HER2+ or TNBC who had 1–2 metastatic SLNs. There were 35 patients who had 1–2 metastatic SLNs in GSH-YCC (discovery cohort) and 97 patients in AMC-SMC (validation cohort). The additional ALN metastasis rates were 31.4% (11 of 35 patients) in GSH-YCC and 28.9% (28 of 97 patients) in AMC-SMC. In patients with 1–2 metastatic SLNs in GSH-YCC, none of the 5 complete MRI responders had additional metastatic non-SLNs, whereas 11 of 30 (36.7%) non-complete MRI responders had additional metastatic non-SLNs. In patients with 1–2 metastatic SLNs in AMC-SMC, additional non-SLN metastases were observed in 21.1% (4 of 19) of complete MRI responders, whereas they were noted in 30.8% (24 of 78) of non-complete MRI responders. We then investigated the additional ALN metastasis rate in patients who had three or more additional negative SLNs as well as 1–2 metastatic SLNs. Among patients with 1–2 SLN metastasis and three or more negative SLNs on frozen examination, none of the nine complete MRI responders had additional ALN metastases. However, 6 of the 34 (17.6%) non-complete MRI responders had additional metastatic ALNs (Table 3).

**Table 3:** Additional axillary lymph node metastasis rate according to radiologic response in patients with 1–2 sentinel lymph node metastases

Cohort	Treatment response	All patients	Number of non-metastatic SLN ≥ 3
GSH-YCC	Complete MRI response	0.0% (0 of 5)	0.0% (0 of 1)
	Non-complete MRI response	36.7% (11 of 30)	20.0% (1 of 5)
	Total	31.4% (11 of 35)	16.7% (1 of 6)
AMC-SMC	Complete MRI response	21.1% (4 of 19)	0.0% (0 of 8)
	Non-complete MRI response	30.8% (24 of 78)	17.6% (6 of 34)
	Total	28.9% (28 of 97)	14.3% (6 of 42)

*SLN* sentinel lymph node, *MRI* magnetic resonance imaging

## Discussion

In this study, the FNRs of SLNB were 0 for the discovery cohort and 7.1% (95% CI 0–16.7) for the validation cohort when HER2+ or TNBC, including primary tumor and metastatic LNs, disappeared completely on breast MRI after NAST. Our findings indicate that SLNB may be a reliable procedure for patients with HER2+ or TNBC node-positive breast cancer, including cN2-3, who show a complete MRI response after NAST. Meanwhile, the FNR of SLNB was > 10% in patients with HER2+ or TNBC who were non-complete MRI responders. Therefore, SLNB alone should be precluded in this subpopulation. Our results suggest that post-NAST breast MRI provides helpful information for determining SLNB-guided axillary surgery or upfront ALND in patients with HER2+ or TNBC.

Although the diagnostic performance of breast MRI outperformed those of other methods in assessing residual disease after NAST [26, 27], MRI-response-guided axillary surgery has not been well-described using a real-world data, including the FNR of SLNB, molecular subtypes, and baseline clinical nodal status. At this point, our data has strength because all our enrolled patients underwent SLNB and subsequent ALND, which allows measurement of the FNR of SLNB. In addition, we classified the patients into two groups on the basis of the subtypes and compared the outcomes of SLNB according to the MRI response in each group. Furthermore, we included patients with cN2-3 who had a high volume of metastatic LNs and were rarely considered as candidates for SLNB in previous studies.

The findings of a previous study by Garcia-Tejedor et al. were similar to ours [28]. Among patients with cN2 breast cancer, they found that nodal pCR after NAST was associated with subtype, clinical and radiologic response, and Ki-67 expression, suggesting that SLNB could be recommended for patients with HER2+ or TNBC with cN2 if there is a complete response. However, they did not depict how they performed axillary surgery, including SLNB; thus, the FNR of SLNB cannot be addressed in these cases.

In contrast to the findings for patients with HER2+ or TNBC, the FNRs of SLNB were > 20% in both complete and non-complete responders of HR+ HER2− breast cancer, suggesting that axillary surgery should not be guided by SLNB, regardless of the radiologic response to node-positive HR+ HER2− breast cancer after NAST. The higher FNR of SLNB in HR+ HER2− breast cancer than that in HER2+ breast cancer or TNBC might be explained by the fact that the pathological axillary response varies by tumor subtype. It is well known that compared with HR+ HER2− breast cancer, HER2+ or TNBC is more likely to achieve axillary nodal pCR [29, 30]; the axillary nodal pCR rate was approximately 20–25% in HR+ HER2− breast cancer and 40–90% in HER2+ or TNBC [31–33]. Another reasoning for our findings is that the accuracy of breast MRI for assessing residual disease after NAST may differ according to the subtype. Residual disease of HR+ HER2− breast cancer after NAST tends to be underestimated than that of HER2+ or TNBC [34–36]. Collectively, this suggests that accurate assessment of radiologic response and prediction of pathologic response are more challenging for HR+ HER2− breast cancer than for other subtypes.

Moreover, we noted that additional ALN metastasis was low in complete MRI responders with HER2+ or TNBC who had 1–2 positive SLNs after NAST. Interestingly, there was no additional ALN metastasis when three or more additional SLNs retrieved during SLNB were tumor-free. Because the number of patients included in the analysis to infer this is too small, multidisciplinary discussions about which patients may safely avoid ALND should continue. Given that the frequency of additional positive ALNs is high in cases with a positive SLN after NAST, regardless of the metastatic volume of SLNs [37], completion ALND is currently recommended. Nevertheless, many researchers wonder whether ALND is inevitable when SLNB identifies low-volume residual disease, such as 1–2 positive SLNs after NAST as a mirror of the ACOSOG Z0011 trial [38]. There is an ongoing trial addressing this issue, Alliance A011202 (NCT01901094), comparing ALND plus radiation versus radiation alone in the setting of a positive 1–2 SLN after NAST [39].

Targeted axillary dissection, which is the removal of SLNs with clipped nodes, substantially reduced the FNR (2.0%) than did SLNB alone (10.6%) [40]. Marking the ALN with radioactive seed (MARI) and tailored axillary surgery procedures, which are similar concepts to less extensive axillary surgery in node-positive breast cancer prior to NAST, showed reasonable FNRs of 6.7% and 2.6%, respectively [41, 42]. Although previous studies did not present the FNRs of these procedures according to the subtypes, we expect that these surgical procedures may improve the accuracy of axillary staging in non-complete MRI responders with HER2+ or TNBC or in those with HR+ HER2− breast cancer. Further studies are needed to verify these issues.

Our study had several limitations. First, this study had patient selection bias because of its retrospective nature. To overcome this limitation, we used a multi-institutional database and validated our results. Second, a central review of breast MRI was not performed in this study although there was a modest inter-method and inter-observer agreement in interpreting MRI images [43]. This may result in slightly different FNR values in non-complete MRI responders with HER2+ or TNBC between the discovery and validation cohorts. Third, the number of complete MRI responders after NAST was relatively low, accounting for < 20% of all patients. This limitation restricts the analysis of the feasibility of SLNB according to MRI response with initial clinical nodal status. However, this limitation may stem from the strict definition of a complete MRI responder, requiring confirmed radiologic complete response in both the breast and axilla. Future research with a larger cohort of patients is warranted to verify and strengthen the robustness of our findings.

SLNB is a reliable surgical method for complete MRI responders with HER+ and TNBC after NAST, even with a high nodal burden at baseline. Conversely, the FNR of SLNB was higher than the acceptable value of 10% in HER2+ or TNBC breast cancer with non-complete MRI response and in HR+ HER2− breast cancer, regardless of treatment response. Therefore, omission of ALND or limited axillary surgery should be carefully considered. By using breast MRI to assess the response to NAST, clinicians may be able to identify HER2+ or TNBC patients who have a low risk of residual axillary disease and who may not require ALND after SLNB, even if they had initially presented with a high nodal burden.

### Supplementary Information

**Additional file 1**. **Fig. S1**. Images of the complete MRI responder (**A**) Breast MRI at pre-neoadjuvant systemic therapy shows a suspicious breast mass with non-mass enhancement in the right breast (solid arrow) and (**B**) multiple enlarged suspicious lymph nodes (more than three at the level I and II) in the right axilla (solid arrow). Matted axillary lymph nodes were detected on physical examination. Accordingly, we defined the clinical nodal stage of this case as cN2. Breast MRI at post-neoadjuvant systemic therapy shows that suspicious features disappear both in the right breast (solid arrow) (**C**) and axilla (solid arrow) (**D**). Abbreviations: MRI, magnetic resonance imaging. **Fig. S2**. Images of the non-complete MRI responder (**A**) Breast MRI at pre-neoadjuvant systemic therapy shows a suspicious breast mass with non-mass enhancement in the right breast (solid arrow), and (**B**) multiple enlarged suspicious lymph nodes (more than three at the level I and II) in the right axilla (solid arrow). Fixed axillary lymph nodes to the underlying structure were detected on physical examination. Accordingly, we defined the clinical nodal stage of this case as cN2. Although the size and enhancement are reduced after neoadjuvant systemic therapy, suspicious features are still observed in both the right breast (solid arrow) (**C**) and axilla (solid arrow) (**D**).

## Data Availability

H.S.Park and S.G.Ahn, corresponding authors, had full access to all the data in the study and takes responsibility for the integrity of the data and the accuracy of the data analysis.
