# Supplementary material for: Outcomes of sentinel node biopsy according to MRI response in an association with the subtypes in cN1–3 breast cancer after neoadjuvant systemic therapy, multicenter cohort study
Source: Breast Cancer Res. 2024 Apr 17;26:66. doi: 10.1186/s13058-024-01807-8 (PMC11022328; doi:10.1186/s13058-024-01807-8)

**Table S1. Baseline characteristics of patients with HR+HER2- breast cancer according to MRI response in the GSH and YCC cohorts**

| Variables | Non-complete MRI responders (N = 182) | Complete MRI responders (N = 25) | Total (N = 207) | *P* value |
| --- | --- | --- | --- | --- |
| Age at diagnosis, y |  |  |  | 0.710 |
| <50 | 109 (59.9) | 14 (56.0) | 123 (59.4) |  |
| ≥50 | 73 (40.1) | 11 (44.0) | 84 (40.6) |  |
| Pathologically confirmed lymph node^a^ |  |  |  | 0.677 |
| Yes | 138 (75.8) | 18 (72.0) | 156 (75.4) |  |
| No | 44 (24.2) | 7 (28.0) | 51 (24.6) |  |
| Removed lymph node^b^ | 16 (10-33) | 13 (10-30) | 15 (10-33) | 0.049 |
| Progesterone receptor |  |  |  | 0.057 |
| positive | 141 (77.5) | 15 (60.0) | 156 (75.4) |  |
| negative | 41 (22.5) | 10 (40.0) | 51 (24.6) |  |
| Histologic grade^b^ |  |  |  | 0.602^c^ |
| 1 or 2 | 150 (93.8) | 16 (100) | 166 (94.3) |  |
| 3 | 10 (6.3) | 0 | 10 (5.7) |  |
| Ki-67^a^ |  |  |  | 0.166 |
| <14% | 117 (69.2) | 12 (54.5) | 129 (67.5) |  |
| ≥14% | 52 (30.8) | 10 (45.5) | 62 (32.5) |  |
| Clinical T stage |  |  |  | 0.755^b^ |
| 1 | 34 (18.7) | 5 (20.0) | 39 (18.8) |  |
| 2 | 114 (62.6) | 14 (56.0) | 128 (61.8) |  |
| ≥3 | 34 (18.7) | 6 (24.0) | 40 (19.3) |  |
| Clinical N stage |  |  |  | > 0.999^b^ |
| 1 | 122 (67.0) | 17 (68.0) | 139 (67.1) |  |
| 2 | 31 (17.0) | 4 (16.0) | 35 (16.9) |  |
| 3 | 29 (15.9) | 4 (16.0) | 33 (15.9) |  |
| Breast operation |  |  |  | 0.451 |
| Breast-conserving surgery | 80 (44.0) | 9 (36.0) | 89 (43.0) |  |
| Mastectomy | 102 (56.0) | 16 (64.0) | 118 (57.0) |  |

^a^Patients with non-pathologically confirmed lymph nodes and radiologically suspicious lymph nodes are included, and nodal staging work-up is assessed using multiple imaging studies.

^b^Missing values

^c^ P-values are obtained using Fisher’s exact test.

Abbreviations: HR, hormone receptor; HER2, human epidermal growth factor receptor 2; MRI, magnetic resonance imaging.

**Figure S1.**

**
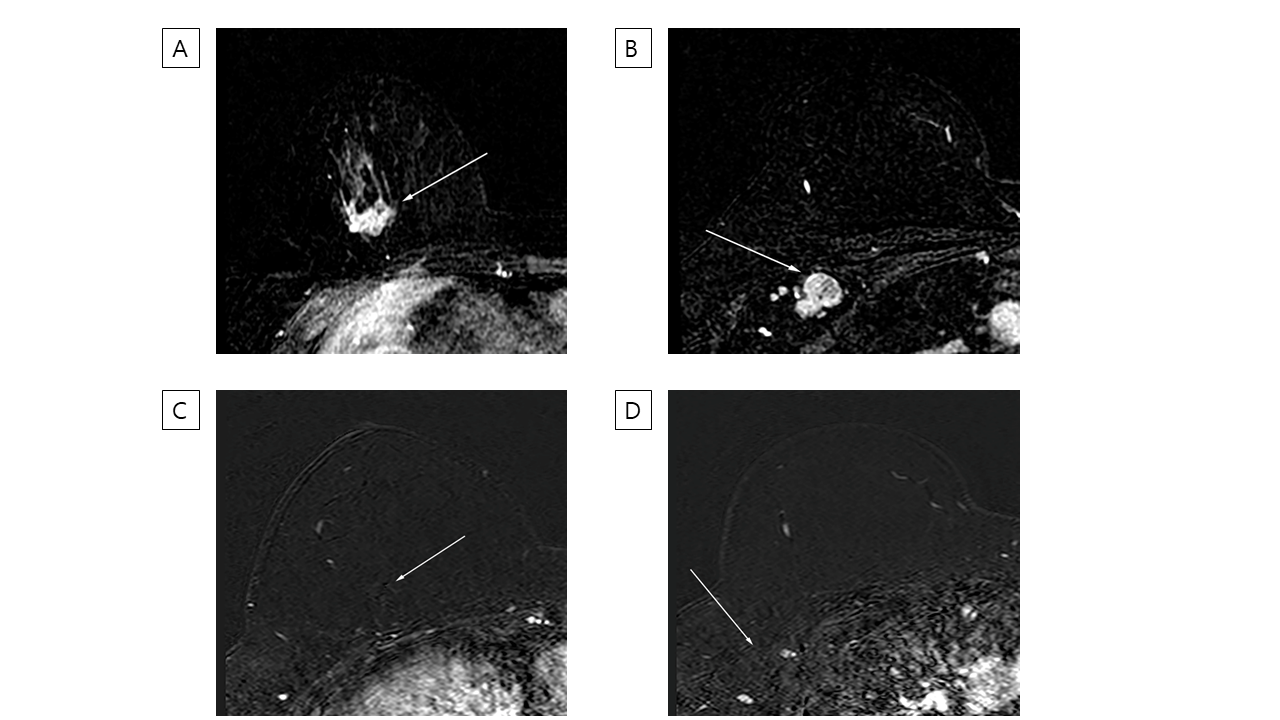
**

**Figure S2.**


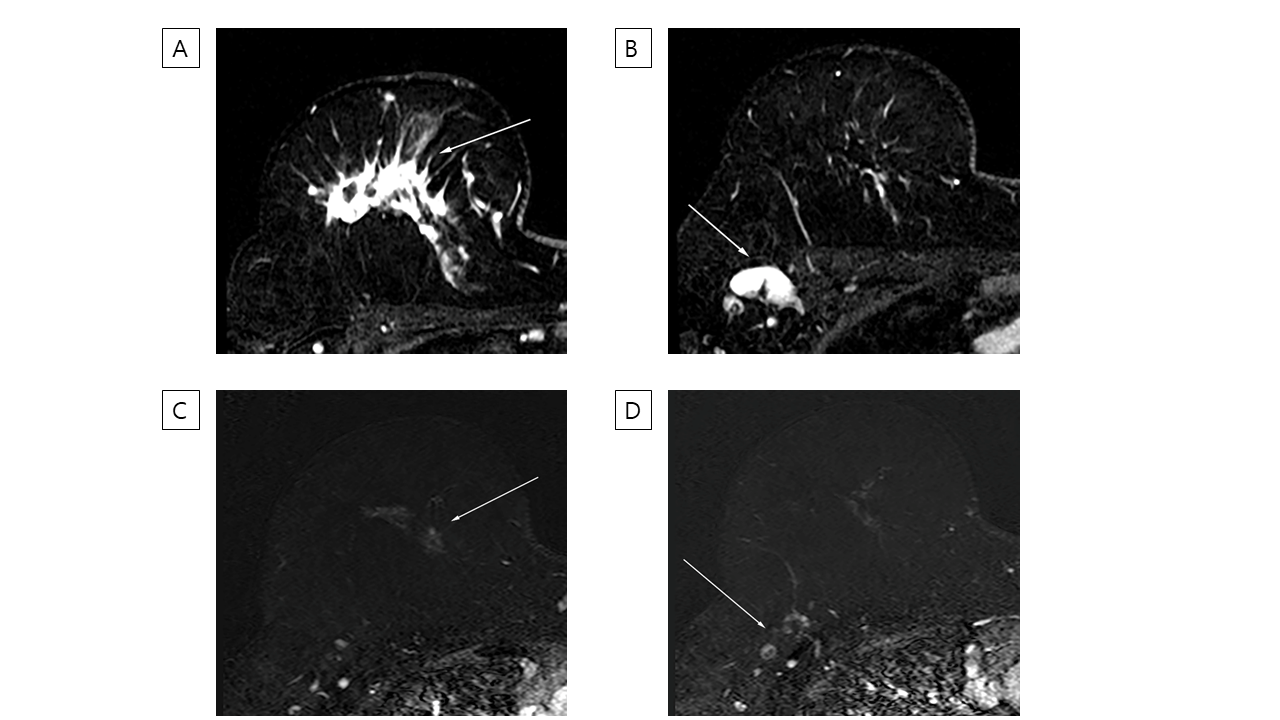

Supplement: Supplementary file 1 — Additional file 1. Fig. S1. Images of the complete MRI responder (A) Breast MRI at pre-neoadjuvant systemic therapy shows a suspicious breast mass with non-mass enhancement in the right breast (solid arrow) and (B) multiple enlarged suspicious lymph nodes (more than three at the level I and II) in the right axilla (solid arrow). Matted axillary lymph nodes were detected on physical examination. Accordingly, we defined the clinical nodal stage of this case as cN2. Breast MRI at post-neoadjuvant systemic therapy shows that suspicious features disappear both in the right breast (solid arrow) (C) and axilla (solid arrow) (D). Abbreviations: MRI, magnetic resonance imaging. Fig. S2. Images of the non-complete MRI responder (A) Breast MRI at pre-neoadjuvant systemic therapy shows a suspicious breast mass with non-mass enhancement in the right breast (solid arrow), and (B) multiple enlarged suspicious lymph nodes (more than three at the level I and II) in the right axilla (solid arrow). Fixed axillary lymph nodes to the underlying structure were detected on physical examination. Accordingly, we defined the clinical nodal stage of this case as cN2. Although the size and enhancement are reduced after neoadjuvant systemic therapy, suspicious features are still observed in both the right breast (solid arrow) (C) and axilla (solid arrow) (D). [file 13058_2024_1807_MOESM1_ESM.docx]
